# α cells use both PC1/3 and PC2 to process proglucagon peptides and control insulin secretion

**DOI:** 10.1126/sciadv.ady8048

**Published:** 2025-09-19

**Authors:** Canqi Cui, Danielle C. Leander, Sarah M. Gray, Kimberley El, Alex Chen, Paul A. Grimsrud, Jessica O. Becker, Austin Taylor, Guo-Fang Zhang, Kyle W. Sloop, C. Bruce Verchere, Andrew N. Hoofnagle, David A. D’Alessio, Jonathan E. Campbell

**Affiliations:** ^1^ Duke Molecular Physiology Institute, Durham, NC, USA.; ^2^ Division of Endocrinology, Department of Medicine, Duke University, Durham, NC, USA.; ^3^ Department of Laboratory Medicine and Pathology, University of Washington, Seattle, WA, USA.; ^4^ Departments of Surgery and Pathology and Laboratory Medicine, Faculty of Medicine, BC Children’s Hospital Research Institute and Centre for Molecular Medicine and Therapeutics, University of British Columbia, Vancouver, BC, Canada.; ^5^ Diabetes, Obesity and Complications, Lilly Research Laboratories, Eli Lilly and Company, Indianapolis, IN, USA.; ^6^ Department of Pharmacology and Cancer Biology, Duke University, Durham, NC, USA.

## Abstract

α cells secrete proglucagon peptides to regulate nutrient metabolism. Recent findings support an α cell–to–β cell axis that is mediated by paracrine signaling through the glucagon receptor and glucagon-like peptide 1 (GLP-1) receptor in β cells. To address which proglucagon peptides stimulate insulin secretion, we developed an assay to quantify levels of GLP-1(7–36)NH_2_. We also generated three transgenic mouse lines that allow α cell-specific, inducible deletion of the genes for the two prohormone convertase enzymes that process proglucagon . Our studies reveal that both mouse and human islets contain GLP-1(7–36)NH2, but glucagon mediates α cell–to–β cell communication in mice. However, in the absence of normal production of glucagon, α cells up-regulate prohormone convertase 1 (PC1/3) to generate GLP-1 and enhance glucose tolerance. Human islets have substantially higher levels of GLP-1 than mice, which positively correlate with rates of insulin secretion. These studies show plasticity in proglucagon processing to support α cell–to–β cell communication.

## Teaser

α cells can generate and secrete both glucagon and GLP-1 with distinct processing enzymes to control metabolism.

## INTRODUCTION

The impairment of metabolic homeostasis that characterizes type 2 diabetes is caused in great part by insufficient insulin secretion to overcome peripheral insulin resistance (*1*). The insulin secretory response is primarily regulated by a combination of metabolic signals derived from glucose and amino acids in combination with activation of G protein (heterotrimeric guanine nucleotide–binding protein)–coupled receptors that amplify these nutrient signals (*2*). Incretins, a term that refers generally to insulinotropic hormones released after eating that activate β cell G protein–coupled receptors, account for up to 70% of postprandial insulin secretion in healthy individuals (*3*). This effect is impaired among people with type 2 diabetes and partially explains their insufficient insulin secretion (*4*). It has long been held that the incretin effect is almost entirely mediated by glucagon-like peptide 1 (GLP-1) and glucose-dependent insulinotropic polypeptide (GIP) secreted from enteroendocrine cells in the intestine (*5*). This model has been revised, in part because of doubts as to whether gut-derived GLP-1, which is secreted in relatively low amounts and is inactivated rapidly once in the circulation, contributes to the incretin effect (*6*, *7*). In this context, GIP has been advanced as the predominant hormone driving the incretin axis (*8*). Nonetheless, on the basis of the results of human and animal studies using experimental GLP-1 receptor (GLP-1R) antagonism or loss of function (*8*–*12*), β cell signaling mediated by this receptor is an essential component of postprandial insulin secretion. Our group and others have shown that the proglucagon peptide ligands for the β cell GLP-1R come from neighboring α cells (*9*, *13*–*16*), an axis we have termed α cell–to–β cell communication (*17*–*19*). Mouse knockout studies have demonstrated that α cell synthesis of proglucagon and β cell expression of the GLP-1R are essential for appropriate quantities of insulin to be secreted in response to a glucose challenge (*13*, *20*). Similarly, antagonism of the GLP-1R in isolated human islets reduces basal and glucose-stimulated insulin secretion (GSIS) (*9*). Collectively, these observations highlight that production of proglucagon peptides by α cells provide essential β cell stimulation through the GLP-1R.

Processing of proglucagon occurs through two distinct enzymes, prohormone convertase 1 (PC1/3) and PC2, with overlapping peptide cleavage sites but specific distributions in islet endocrine cells (*21*). In mouse islets, both hormone convertase genes, *Pcsk1* (encoding for PC1/3) and *Pcsk2* (encoding for PC2), are expressed in β and δ cells, while α cells produce predominantly *Pcsk2* to cleave glucagon, but not GLP-1, from proglucagon (*22*). In contrast, PC1/3 is highly expressed in intestinal proglucagon-producing L cells, which results in the production and secretion of GLP-1, GLP-2, and oxyntomodulin but little glucagon (*23*). Mice with global knockout of *Pcsk1* display dwarfism along with defects in the processing of several neuroendocrine peptides including GLP-1 (*24*, *25*). Mice with a global deletion of *Pcsk2* have impaired the processing of proinsulin, prosomatostatin, and proglucagon; these animals have reduced fasting blood glucose, small glycemic excursions in response to a glucose tolerance test, and α cell hyperplasia (*26*).

The specificity of α and L cells to produce either glucagon or GLP-1 is likely not absolute, and immunoreactive GLP-1 has been detected in both human and murine islets (*27*–*32*). Moreover, several studies have demonstrated that α cells increase GLP-1 production in response to metabolic stress (*33*, *34*). While the primary physiological role attributed to glucagon is as a counterregulatory factor that protects against hypoglycemia by stimulating hepatic glycogenolysis (*17*), it is also insulinotropic and secreted in response to mixed nutrient meals, a setting in which stimulation of insulin secretion would contribute to the incretin effect. Although both glucagon and GLP-1 can activate the GLP-1R to stimulate insulin secretion (*9*, *15*, *35*, *36*), GLP-1 is ~300× more insulinotropic than glucagon in mouse islets (*9*). Thus, it has been proposed that differential proglucagon processing may be a mechanism to enhance α cell–to–β cell communication in settings where greater insulin secretion is needed (*7*). However, because α cell GLP-1 production is at least two orders of magnitude less than glucagon (*9*), there has been skepticism that sufficient amounts are produced to have meaningful activity (*15*, *37*). The limited quantities of α cell GLP-1 also pose a challenge for the sensitivity and specificity of most immunoassays.

We hypothesized that α cells have sufficient PC1/3 to process proglucagon into GLP-1 as an adaptation to metabolic demand. To test this hypothesis, we created mouse lines with conditional α cell deletion of *Pcsk1*, *Pcsk2*, and both. Our findings demonstrate that both glucagon and GLP-1 can serve as the primary mediator of α cell–to–β cell communication in mice and reinforce previous demonstrations that proglucagon peptides are essential for normal β cell function. The islet GLP-1 content is substantially greater in human samples and correlates with ex vivo GSIS.

## RESULTS

### Development of an assay to measure GLP-1(7–36)NH_2_

**Fig. 1.**
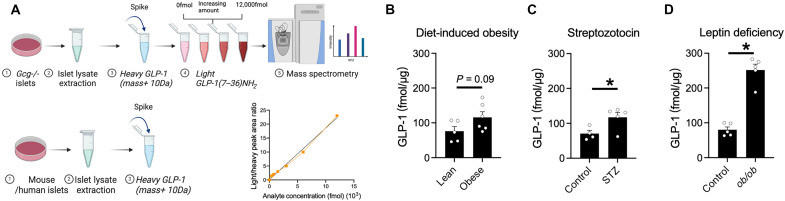
Quantifying active GLP-1 in mouse islets. (**A**) MS-based assay for quantifying levels of active GLP-1 [GLP-1(7–36)NH_2_] in islets. A standard curve is generated using islets from *Gcg^−/−^* mice, where constant amounts of heavy isotope–labeled GLP-1(7–36)NH_2_ and varied amounts of unlabeled (light) GLP-1(7–36)NH_2_ are added to islet lysates. The same level of heavy isotope–labeled GLP-1(7–36)NH_2_ is added to unknown islet samples. The ratio of light:heavy GLP-1(7–36)NH_2_ is plotted on the standard curve to quantify the amount of GLP-1(7–36)NH_2_ in the unknown samples. (**B** to **D**) Islet GLP-1(7–36)NH_2_ levels in different models of metabolic stress, including (B) diet-induced obesity, where wild-type C57BL/6J mice are fed a 60% high-fat diet for 14 weeks (*n* = 5 and 6); (C) hyperglycemia, where wild-type C57BL/6J mice are given STZ to induce β cell death and hypoinsulinemia (*n* = 5); and (D) leptin deficiency–induced obesity and hyperglycemia (*n* = 5). *P < 0.05 as indicated. Data are shown as the means ± SEM. Statistical analysis was done with Student’s unpaired *t* test.

Accurate measurement of GLP-1 with conventional immunoassay methods has limitations for distinguishing the bioactive, intact GLP-1 fragments [GLP-1(7–37) and GLP-1(7–36)NH_2_] from other peptides derived from proglucagon (*38*). To overcome this limitation, we developed a mass spectrometry (MS)–based assay to specifically quantify GLP-1(7–36)NH_2_. Islet extracts from *Gcg^−/−^* mice (*6*) were used to provide a tissue matrix devoid of proglucagon peptides to which known amounts of synthetic GLP-1(7–36)NH_2_ (light) could be added to generate a standard curve for quantification (Fig. 1A). GLP-1 with a modified phenylalanine at position 28 containing stable heavy isotopes, providing a 10-Da increase in mass (heavy GLP-1), was used as a standard, and the ratio of light:heavy GLP-1 in unknown samples was read against the standard curve as a measure of the amount of peptide. Using this assay, we measured levels of GLP-1(7–36)NH_2_ in extracts of C57BL6/J mouse islets at levels of ~50 to 250 fmol/μg tissue (Fig. 1, B to D). Islets from mice with obesity from a high-fat diet or leptin deficiency and animals given a single dose of streptozotocin (STZ) had higher islet content of GLP-1 than controls. In healthy C57/BL6 mice, the amounts of GLP-1(7–36)NH_2_ in islets were substantially lower than in extracts of intestinal tissue (fig. S1A).

### Deletion of α cell PC1/3 reduces islet GLP-1

**Fig. 2.**
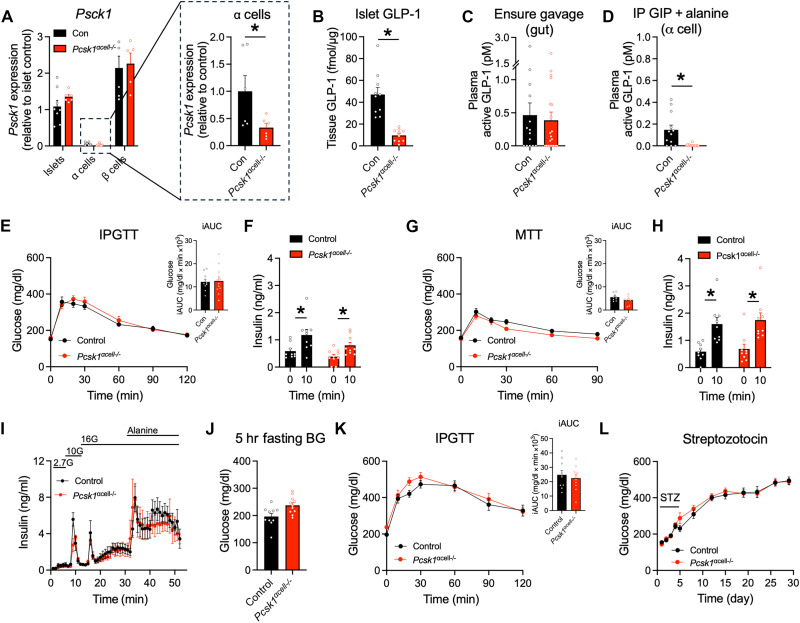
Deletion of *Pcsk1* in α cells does not affect β cell function or glucose tolerance. (**A**) Relative mRNA levels of *Pcsk1* in whole islets and enriched populations of α and β cells (*n* = 6 to 8). Data are normalized to control values of whole islets. The inset shows the data for the α cells only, normalized to control islets. (**B**) GLP-1(7–36)NH_2_ content of islet lysates determined by MS (*n* = 11). (**C**) Plasma levels of active GLP-1 measured by ELISA following oral gavage with Ensure after an overnight fast (*n* = 14 and 20). (**D**) Plasma levels of active GLP-1 measured by ELISA following intraperitoneal (IP) injection of GIP (4 nmol/kg) and alanine (0.325 g/kg) after a 5-hour fast (*n* = 12 and 17). (**E** and **F**) Glucose tolerance (E) and plasma insulin (F) after intraperitoneal injection of glucose (1.5 g/kg) (*n* = 10 and 11). (**G** and **H**) Glucose tolerance (G) and plasma insulin (H) after oral gavage of a mixed nutrient stimulus (*n* = 9 and 9). (**I**) Islet perifusion to measure rates of insulin secretion in response to difference concentrations of glucose alone and with alanine (3 mM) (*n* = 5 and 5). (**J** and **K**) Fasting glycemia (J) (*n* = 10) and intraperitoneal glucose tolerance (K) in diet-induced obese mice fed a 60% high-fat diet for 14 weeks (*n* = 10 and 9). hr, hour. (**L**) Ambient glycemia following 5-day treatment with multiple, low-dose (50 mg/kg) STZ (*n* = 10 and 11). *P < 0.05 as indicated. Data are shown as the means ± SEM. Statistical analysis was done with Student’s unpaired *t* test [(A) to (D)] or analysis of variance (ANOVA) with post hoc analysis [(E) to (L)].

With the demonstration that mouse islets had detectable GLP-1 content and that animals with obesity or β cell damage had increased GLP-1 levels, we hypothesized that the loss of α cell GLP-1 would decrease α cell–to–β cell communication, resulting in impaired insulin secretion and glucose intolerance. To test this hypothesis, mice were created that had an inducible deletion of *Pcsk1* in α cells (*Pcsk1*^αcell−/*−*^) to reduce PC1/3 levels and production of intact, active GLP-1 (*21*). *Pcsk1*^αcell−/−^ and control mice were given tamoxifen at 8 weeks of age to induce Cre-mediated recombination, and cohorts were studied a minimum of 4 weeks later. This time frame allowed for the turnover of intestinal enteroendocrine cells from stem cells, ensuring normal proglucagon processing in L cells. In cohorts of knockout and control mice, the expression of *Pcsk1* in the enriched population of α cells was markedly less than in whole islets or enriched populations of β cells (Fig. 2A). The levels of *Pcsk1* in the α cells of *Pcsk1*^αcell−/*−*^ mice were ~60% lower than those of controls without changes in β cell or gut *Pcsk1* expression (Fig. 2A and fig. S1B). This corresponded to an ~80% reduction of α cell GLP-1 content in islets from *Pcsk1*^αcell−/−^ mice measured by MS (Fig. 2B) and in pancreas extracts measured by enzyme-linked immunosorbent assay (ELISA) (fig. S1C). The amounts of active GLP-1 from intestinal extracts were not different in *Pcsk1*^αcell−/−^ and control mice (fig. S1D). To test the impact of this intervention on circulating levels of active GLP-1, mice were given Ensure by oral gavage as an enteroendocrine cell stimulus or intraperitoneal injection of GIP and alanine as an α cell stimulus (*20*). Oral nutrients produced an equivalent rise in plasma GLP-1 in control and *Pcsk1*^αcell−/−^ mice (Fig. 2C), while intraperitoneal GIP and alanine induced a rise in plasma GLP-1 only in the control mice (Fig. 2D). These findings support an important functional reduction in α cell GLP-1 production as a result of the induced deletion of *Pcsk1*.

No differences in body weight, glycemia, insulin concentrations, and GLP-1 or glucagon levels were observed between the *Pcsk1*^αcell−/−^ and control mice (fig. S1, E to K). Glucose tolerance and the insulin response to intraperitoneal glucose or a gavage of glucose or mixed nutrients were also similar between the groups (Fig. 2, E to H, and fig. S1, L and M). In isolated islets, rates of insulin secretion were similar between control and *Pcsk1*^αcell−/−^ islets when challenged with high glucose alone or with the addition of alanine to augment α cell–to–β cell communication (Fig. 2I). Together, these results indicate that targeted reduction of α cell GLP-1 did not affect the β cell function or glucose tolerance in lean mice.

Given that obese or STZ-treated mice had elevated islet GLP-1 concentrations, we tested whether metabolic stress would unmask a systemic phenotype in the *Pcsk1* knockout animals (Fig. 1, B to D). *Pcsk1*^αcell−/−^ and control mice were fed a high-fat diet for 14 weeks to induce obesity; this intervention caused no differences in body weight, ambient glycemia, fasting glycemia, or glucose tolerance following either intraperitoneal glucose or an oral mixed nutrient stimulus (Fig. 2, J and K, and fig. S1, N to P). To enhance the level of metabolic stress, we challenged obese mice with multiple, low-dose STZ. However, both control and *Pcsk1*^αcell−/−^ mice became equally diabetic after this treatment (Fig. 2L). Thus, on the basis of these two methods of inducing metabolic stress, the loss of α cell GLP-1 had little impact on glucose regulation.

### Deletion of α cell PC2 reduces islet glucagon

**Fig. 3.**
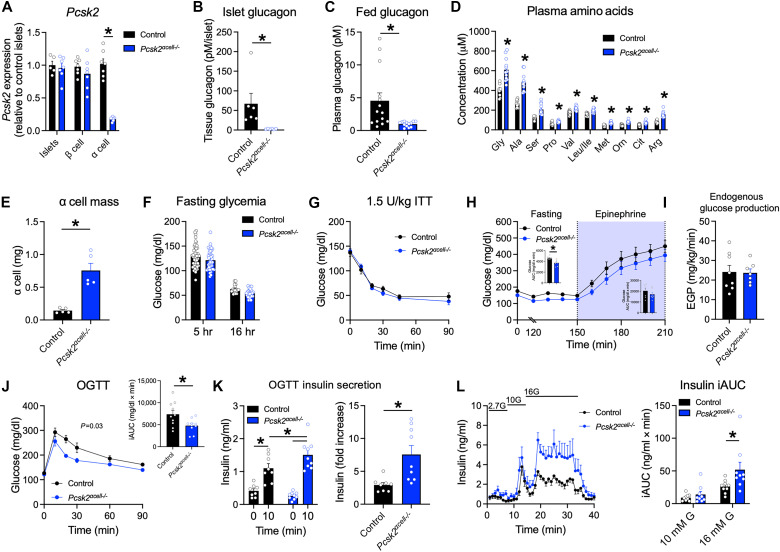
Deletion of *Pcsk2* in α cells improves the β cell function. (**A**) *Pcsk2* expression in whole islets and enriched populations of α and β cells (*n* = 5 to 7). Data are normalized to the value of whole islets from controls. (**B** and **C**) Glucagon levels in (B) islets (*n* = 6) and (C) plasma (*n* = 14) measured by MS. (**D**) Fasted plasma amino acid levels (*n* = 10, and 12). (**E**) α cell mass in 20-week-old mice (*n* = 5). (**F**) Fasted glycemia (*n* = 30 to 48). (**G**) Insulin tolerance test (ITT; 1.5 U/kg) in 5 hour–fasted mice (*n* = 7). (**H** and **I**) Glucose turnover in 5 hour–fasted mice. (H) Glycemia during the fasted period (0 to 150 min) and following infusion of epinephrine (5 μg kg^−1^ min^−1^) (*n* = 8 and 7). (I) Endogenous glucose production (EGP) measured during the final 30 min of the fasting period (*n* = 8 and 7). (**J** and **K**) Glycemia (J) and plasma insulin (K) during an OGTT (1.5 g/kg) in 5 hour–fasted mice (*n* = 10). (**L**) Insulin secretion in perifused islets (*n* = 9). Incremental AUCs (iAUCs) are shown for 10G (8 to 16 min) and 16G (16 to 32 min) periods. *P < 0.05 as indicated. Data are shown as the means ± SEM. Statistical analysis was done with Student’s unpaired *t* test [(A) to (E) and (H) to (K)] or ANOVA with post hoc analysis [(G), (H), and (J) to (L)].

The insulinotropic actions of glucagon have been known for decades (*17*, *39*), and recent work supports the importance of its intraislet signaling in α cell–to–β cell communication (*9*, *10*, *15*, *16*, *20*, *35*). To target α cell glucagon production, we generated an inducible model of *Pcsk2* deletion in α cells (*Pcsk2*^αcell−/−^ mice), with a similar strategy for knocking out PC2 that we used to eliminate PC1/3. The expression levels of *Pcsk2* were similar in extracts of whole islets compared to extracts obtained from individual enriched populations of β or α cells (Fig. 3A and fig. S2A). *Pcsk2* was ~80% reduced in the enriched α cells of *Pcsk2*^αcell−/−^ mice (Fig. 3A), which corresponded to substantial reduction of islet glucagon content (Fig. 3B) and ambient levels of circulating glucagon (Fig. 3C). Control and *Pcsk2*^αcell−/−^ mice had similar levels of intestinal and brain expression of *Gcg* and *Pcsk2*, intestinal GLP-1 content, and β cell mass (fig. S2, B to D). The reduction in circulating glucagon corresponded to an increase in plasma amino acids (Fig. 3D) and α cell mass (Fig. 3E and fig. S3), two features that are universally seen across models of impaired glucagon activity (*40*–*43*). Collectively, these results show that the induced deletion of *Psck2* was sufficient to cause major alterations in systemic processes regulated by glucagon.

The prominent decrease in plasma glucagon concentrations in *Pcsk2*^αcell−/−^ mice raised the question of increased susceptibility to hypoglycemia. These animals had reduced expression of gluconeogenic genes in liver extracts but no changes in genes that regulate lipid or ketone metabolism (fig. S4A). In keeping with the reduced gluconeogenic gene expression, the knockout animals had a muted glycemic response to exogenous alanine (fig. S4B). The glycemic response to exogenous glycerol was similar between control and *Pcsk2*^αcell−/−^ mice (fig. S4C), suggesting a specific reduction in amino acid conversion to glucose rather than a general reduction in gluconeogenic capacity. Administration of exogenous epinephrine produced a similar glucose response in both groups, suggesting comparable glycogenolytic capacity (fig. S4D). There was no difference in blood glucose concentrations after 5 or 16 hours of fasting between *Pcsk2*^αcell−/−^ and control mice (Fig. 3F), and the rate of onset and the depth of hypoglycemia in response to exogenous insulin (Fig. 3G) were similar. To directly assess basal endogenous glucose production, we performed glucose turnover studies under fasting conditions followed by stimulation with epinephrine as a positive control (Fig. 3H). While glycemia was moderately lower in *Pcsk2*^αcell−/−^ mice during both conditions, endogenous glucose production was similar to controls (Fig. 3I). Overall, there was no clear evidence of an impaired counterregulatory response and minimal differences in hepatic glucose production in the absence of glucagon caused by *Pcsk2* deletion.

We next turned our attention to α cell–to–β cell communication and whether lack of glucagon production influences glucose homeostasis during hyperglycemia. Compared to controls, *Pcsk2*^αcell−/−^ mice had better glucose tolerance during an oral glucose tolerance (OGTT) (Fig. 3J), along with enhanced insulin secretion (Fig. 3K). These results were recapitulated when mice were challenged with an intraperitoneal glucose tolerance test (IPGTT) or meal tolerance test (MTT) (fig. S5, A to D). Islet perifusion experiments showed enhanced GSIS in islets from *Pcsk2*^αcell−/−^ mice compared with control islets (Fig. 3L), indicating that at least a portion of the superior in vivo insulin response was due to factors inherent in the islets. Thus, elimination of α cell production of glucagon did not impair α cell–to–β cell communication but rather enhanced insulin secretion.

### Deletion of PC2 increases PC1/3 expression and islet GLP-1 content

**Fig. 4.**
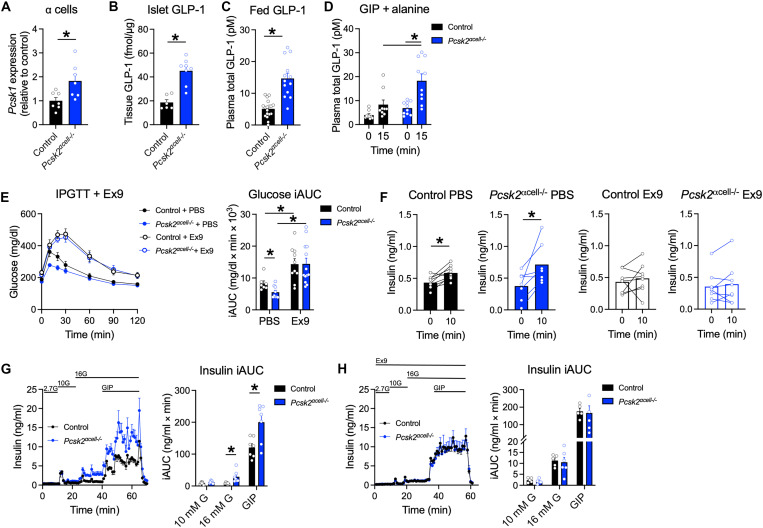
Deletion of *Pcsk2* in α cells increases *Pcsk1* expression and GLP-1 production. (**A**) *Pcsk1* expression in enriched population of α cells (*n* = 7). (**B**) Islet GLP-1(7–36)NH_2_ measured by MS (*n* = 6 and 8). (**C**) Fed total GLP-1 levels in plasma (*n* = 20 and 13). (**D**) Plasma total GLP-1 levels in 5 hour–fasted mice injected with GIP (4 nmol/kg) and alanine (0.325 g/kg) (*n* = 10). (**E** and **F**) Glycemia (E) and plasma insulin levels (F) during an IPGTT (1.5 g/kg) +/− Ex9 (*n* = 7 to 13). (**G** and **H**) Insulin secretion in perifused islets during (G) control conditions (*n* = 7) or (H) with 1 μM Ex9 (*n* = 5 and 6). *P < 0.05 as indicated. Data are shown as the means ± SEM. Statistical analysis was done with Student’s unpaired *t* test [(A) to (C) and (F)] or ANOVA with post hoc analysis [(D), (E), (G), and (H)].

The unexpected result that *Pcsk2*^αcell−/−^ mice have enhanced insulin secretion led us to speculate that there may be a compensatory increase in PC1/3 expression and production of GLP-1 to facilitate α cell–to–β cell communication. In support of this, we found that islets from *Pcsk2*^αcell−/−^ mice have elevated α cell *Pcsk1* expression and an increase in islet GLP-1 content (Fig. 4, A and B). In vivo, this translated to increased plasma concentrations of GLP-1 under both ambient conditions (Fig. 4C) and in response to the α cell stimulus GIP and alanine (Fig. 4D). To determine whether the increased GLP-1 production in the α cells of *Pcsk2*^αcell−/−^ mice accounts for the improved glucose tolerance and insulin secretion, we undertook a series of experiments to interrogate α cell–to–β cell communication. First, we performed IPGTT experiments in the presence or absence of exendin(9–39) (Ex9) to determine the role of GLP-1R signaling (*9*, *13*, *20*). In the absence of Ex9, *Pcsk2*^αcell−/−^ mice again showed improved glucose tolerance and enhanced insulin secretion (Fig. 4E). However, the difference between control and *Pcsk2*^αcell−/−^ mice was abolished by Ex9 (Fig. 4E). The enhanced insulin responses in perifused islets from *Pcsk2*^αcell−/−^ mice (Fig. 4G) were also eliminated by Ex9 (Fig. 4H). Even when α cell–to–β cell communication was enhanced with alanine stimulation of α cells, insulin secretion by perifused islets from *Pcsk2*^αcell−/−^ mice was completely blocked by Ex9 (fig. S5E) but not a glucagon receptor antagonist (fig. S5F). Our previous work in this area showed that α cell–to–β cell communication mediated by glucagon is partly attenuated by the antagonism of the glucagon receptor (*9*, *13*). The observation that islets from *Pcsk2*^αcell−/−^ mice are only sensitive to Ex9 and not glucagon receptor antagonism indicates that GLP-1, and not glucagon, is the ligand supporting α cell–to–β cell communication in these animals.

### Deletion of both PC1/3 and PC2 inhibits α cell–to–β cell communication

**Fig. 5.**
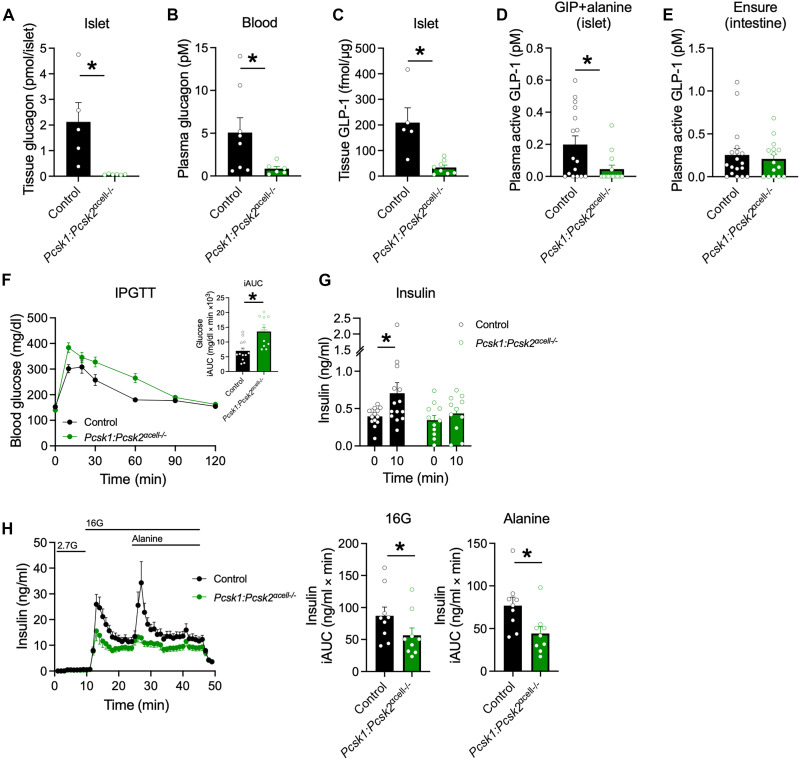
Loss of both *Pcsk1* and *Pcsk2* in α cells leads to impaired α cell–to–β cell function. (**A** and **B**) Glucagon concentrations in (A) islets and (B) plasma measured by MS (*n* = 6 to 8). (**C**) Islet GLP-1(7–36)NH_2_ concentrations measured by MS (*n* = 5 and 9). (**D** and **E**) Plasma active GLP-1 concentrations after (D) intraperitoneal injection of GIP (4 nmol/kg) and alanine (0.325 g/kg) after a 5-hour fast (*n* = 16) or (E) oral gavage with Ensure after an overnight fast (*n* = 18 and 15). (**F** and **G**) Glycemia (F) and plasma insulin levels (G) during an IPGTT (1.5 g/kg) (*n* = 14 and 11). (**H**) Insulin secretion in perifused islets (*n* = 7). iAUCs are shown for 16G (8 to 20 min) and alanine (24 to 40 min). *P < 0.05 as indicated. Data are shown as the means ± SEM. Statistical analysis was done with Student’s unpaired *t* test [(A) to (F) and (H)] or ANOVA with post hoc analysis (G).

To confirm the importance of compensatory increases in α cell PC1/3 and GLP-1 to the improved glucose tolerance observed in *Pcsk2*^αcell−/−^ mice, we generated a line with α cell deletion of both *Pcsk1* and *Pcsk2* (*Pcsk1:Pcsk2*^αcell−/−^ mice). The glucagon content was markedly reduced in both the islet and plasma from *Pcsk1:Pcsk2*^αcell−/−^ mice (Fig. 5, A and B), with a corresponding increase in α cell mass and circulating amino acids (fig. S6, A and B). Islet GLP-1 was also greatly reduced in *Pcsk1:Pcsk2*^αcell−/−^ mice (Fig. 5C), which led to reductions in plasma GLP-1 in response to GIP + alanine (Fig. 5D) but not an enteral stimulus (Ensure; Fig. 5E). Reduction in both islet glucagon and GLP-1 led to impaired glucose tolerance when challenged with an IPGTT (Fig. 5F), manifested by reduced insulin secretion both in vivo (Fig. 5G) and in perifused ex vivo islets (Fig. 5H). *Pcsk1:Pcsk2*^αcell−/−^ mice had improved glucose tolerance when challenged with an OGTT (fig. S6C) but no differences in glucose tolerance in response to an MTT (fig. S6D). Our previous work demonstrated the requirement of β cell GLP-1R signaling for glucose tolerance during an IPGTT, a shift in reliance to the β cell GIP receptor (GIPR) signaling during an OGTT, and the necessity of both systems during an MTT (*20*). From this and on the basis of the current results across various physiological tests, we hypothesized that *Pcsk1:Pcsk2*^αcell−/−^ mice would be more sensitive to GIPR agonism, aligning with our previous observations in models of impaired α cell–to–β cell communication (*9*, *44*). Exogenous GIP had a greater effect during an IPGTT to lower glucose in *Pcsk1:Pcsk2*^αcell−/−^ mice compared to controls (fig. S6E), and a GIPR antagonist ameliorated the differences in glucose tolerance during an OGTT (fig. S6F). Last, perifused islets from *Pcsk1:Pcsk2*^αcell−/−^ mice responded with greater insulin secretion when stimulated with GIP compared to controls (fig. S6G). Collectively, these data illustrate that reducing the production of proglucagon products from α cells leads to impaired α cell–to–β cell communication, reductions in insulin secretion, and glucose intolerance across a variety of physiological interventions, which is masked to a degree by the enhanced sensitivity to GIP.

### Islet GLP-1 levels vary across human islets and are positively correlated with GSIS

**Fig. 6.**
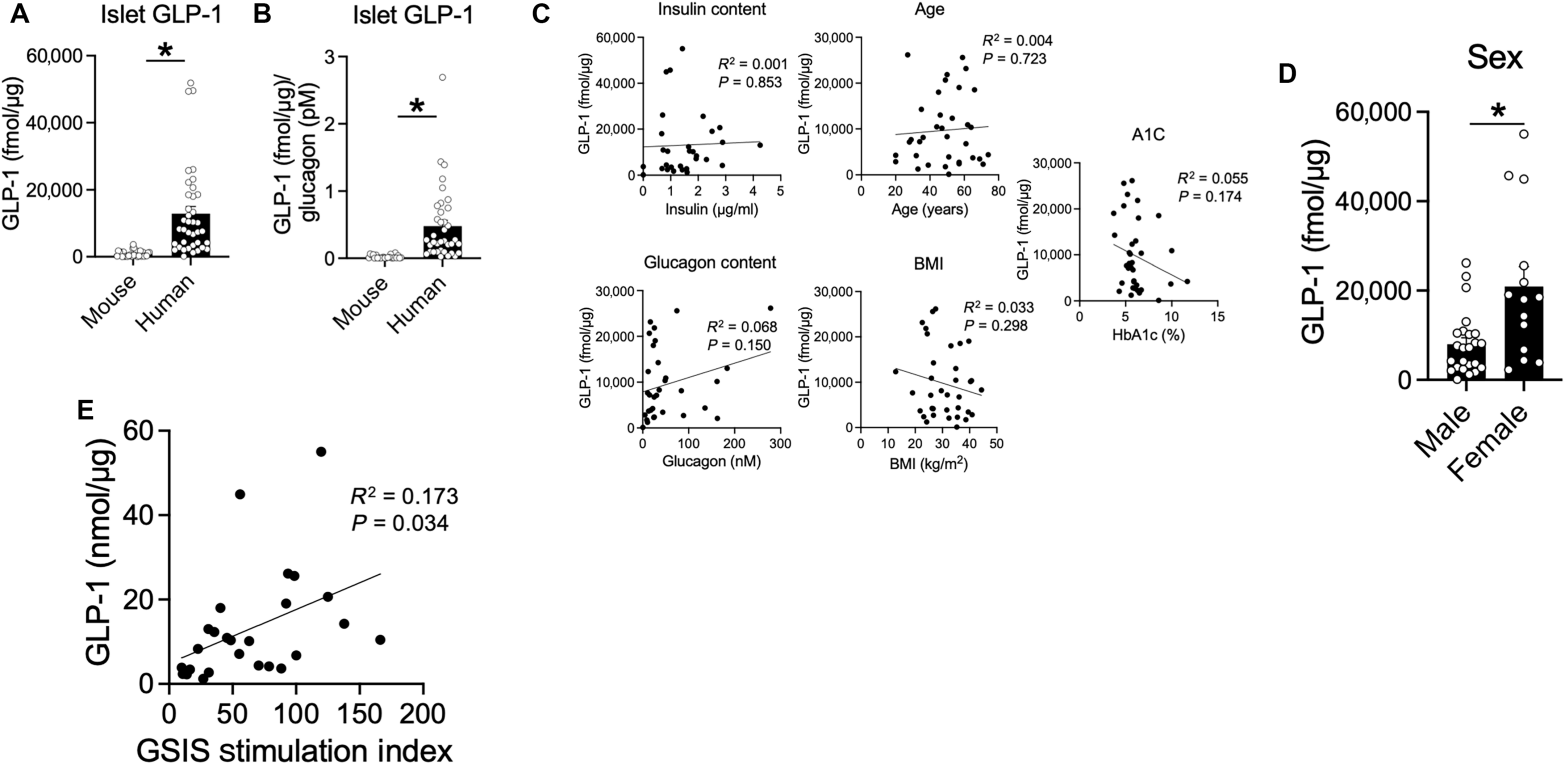
Human islets produce active GLP-1 levels that correlated with rates of GSIS. (**A** and **B**) Islet GLP-1(7–36)NH_2_ content measured by MS and expressed as (A) GLP-1 concentration or (B) normalized to glucagon content (*n* = 23 and 38). (**C** and **D**) Scatterplots between GLP-1(7–36)NH_2_ concentrations and islet or donor characteristics (*n* = 38). (**E**) Scatterplot showing the correlation between islet GLP-1(7–36)NH_2_ concentrations and the insulin secretion index (2.7 to 16 mM glucose) (*n* = 31). *P < 0.05 as indicated. Data are shown as the means ± SEM. Statistical analysis was done with Student’s unpaired *t* test [(A) and (B)] or a regression analysis to calculate the Pearson correlation coefficient [(C) and (D)].

To evaluate a potential role for α cell GLP-1 in humans, we used our MS assay to assess GLP-1(7–36)NH_2_ content across 38 individual sets of human islets. We found that the GLP-1(7–36)NH_2_ content was substantially higher compared to mouse islets (Fig. 6A), even accounting for the higher composition of α cells in humans by normalizing to glucagon content (Fig. 6B). The 38 sets of human islets that were assayed spanned a range of donor characteristics, including age, sex, body mass index (BMI), and hemoglobin A1C (HbA1C), and included islets mostly from donors without diabetes but from several donors who had antemortem type 1 or type 2 diabetes (table S2). The wide variation in islet GLP-1 content in this dataset (Fig. 6A) prompted us to seek associations using simple correlation to the donor characteristics available to us; in these analyses, there were no significant associations observed (Fig. 6C). Of note, islets from female donors in this cohort did have higher levels of islet GLP-1(7–36)NH_2_ than male donors (Fig. 6D). Last, in a subset of islets that had been tested for insulin secretion at the time of isolation, there was a positive correlation between the GSIS stimulation index and the level of islet GLP-1(7–36)NH_2_ content (Fig. 6E). These results indicate that α cell production of GLP-1 may be of greater importance for α cell–to–β cell communication in humans.

## DISCUSSION

In these studies, we accomplished two independent but related goals. The first was to design and implement an assay that would allow for accurate and specific quantitation of bioactive GLP-1 in rodent and human islets. We developed and validated an MS-based assay and demonstrated with some surety that both mouse and human islets can process proglucagon to GLP-1(7–36)NH_2_, a bioactive form of GLP-1 that can regulate insulin secretion in β cells. The second goal was to generate and test mouse models to distinguish the contributions of glucagon and GLP-1 in α cell–to–β cell communication, a refinement of emerging studies supporting proglucagon products in paracrine regulation of the β cell function. The results of these studies show that under most conditions, glucagon is the predominant mediator of α cell–to–β cell communication in mice. However, in response to deletion of PC2, α cells adapt to production of GLP-1 that enhances insulin secretion and glucose tolerance beyond the normative standard of control mice. Deletion of both PC1/3 and PC2 removes the major insulinotropic proglucagon peptides and recapitulates our previous work, demonstrating that these are necessary for α cell–to–β cell communication and normal glycemic regulation (*9*). Last, our experiments in human islets produce a provocative correlation between islet GLP-1 content and insulin secretion in response to glucose, a fundamental parameter in glucose regulation. This raises the possibility that islet GLP-1 may be more influential in humans than rodents, a conjecture supported by the substantially higher islet GLP-1 content of their islets. Future work is required to extend these correlations, ideally in both primary human islets ex vivo and studies of human subjects.

There has been a running debate over several decades as to whether α cells produce meaningful amounts of PC1/3 and active GLP-1 to contribute to the β cell function. This controversy was heightened by observations that STZ-induced β cell damage (*31*) or reduction in glucagon signaling (*45*, *46*) increased islet PC1/3 expression and GLP-1 content. These findings emerged at a time when several groups were demonstrating the importance of β cell GLP-1R activity for physiological insulin secretion and glucose tolerance (*11*, *47*), while other investigators raised doubts that circulating GLP-1 from intestinal enteroendocrine cells could provide sufficient ligand to drive this effect (*6*, *7*). Together, these reports advanced the idea that a local paracrine system existed by which GLP-1 from α cells could regulate β cell GLP-1R (*30*). Further evidence in support of this came from reports showing that β cells depend on proglucagon-derived peptides from α cells (*32*) and that α cells set the glycemic set point of different species by regulating insulin secretion (*14*). These hypotheses were supported by additional studies showing that α cells increase the production of GLP-1 in response to treatment with a GLP-1R agonist or bariatric surgery (*29*, *48*, *49*) and that subpopulations of α cells contain PC1/3 (*28*, *33*). A major question underlying this body of research was a lack of consistency as to whether the GLP-1 species detected in α cells was bioactive [GLP-1(7–36)NH_2_ or GLP-1(7–37)] or another proglucagon fragment that does not have activity at the GLP-1R. It has been suggested that the most abundant GLP-1–like peptide produced by α cells is GLP-1(1–37) (*15*), which does not activate the GLP-1R. To address this, we developed an MS-based assay that quantifies the levels of GLP-1(7–36)NH_2_. Using this assay, we show with reasonable certainty that both mouse and human islets produce some level of bioactive GLP-1. In mice, we show that the levels of GLP-1 can increase in response to various metabolic stressors. Human islets have substantially more GLP-1 compared to mouse islets, even when accounting for the greater percentage of α cells in human islets. These data provide quantitative data to answer the question of whether there is production of bioactive GLP-1 in the islet.

Having established that α cells make and secrete GLP-1, it then became important to determine the functional relevance to insulin secretion and glucose tolerance. Because of the sensitivity of our MS GLP-1 assay, we were able to demonstrate that by targeting *Pcsk1* for deletion, we substantially reduced the low levels of α cell GLP-1 present in wild-type mice. Because this knockdown did not affect glucagon levels, we could determine the impact of reduced α cell GLP-1 in isolation. Traub and colleagues (*32*) used a similar general strategy but with a mouse model that had constitutively active Cre recombinase. These animals had persistent Cre activity in both α cells and L cells, and although there was some reduction of islet *Psck1,* plasma and intestinal GLP-1 was not diminished. In this model, there was a modest worsening of glucose tolerance with PC1/3 deletion in the presence of metabolic stress induced by high-fat diet feeding with STZ treatment. These results differ from what we report here and are likely explained by differences in the animal models and the degree of metabolic stress. The conclusion from our studies of *Pcsk1*^αcell−/−^ mice was that islet GLP-1 is dispensable for glucose regulation in healthy, obese, or diabetic mice. The strength of this conclusion is based on our well-validated model and amply powered ex vivo and in vivo experiments. While it is possible that our mice did not receive an adequate metabolic challenge to drive PC1/3 production and illuminate a role for α cell GLP-1, we used methods that are standard for inducing murine diabetes. Rather, we believe that our results are compatible with plasticity in α cell function that allows mice with sufficient production of glucagon to maintain normal glucose regulation.

The minimal phenotype arising from the depletion of islet GLP-1 turned our attention to glucagon as the primary mediator of α cell–to–β cell communication. Targeting *Pcsk2* with an inducible mechanism eliminated glucagon production of α cells while allowing intestinal proglucagon processing to recover. Knockdown of PC2 was apparent in the low islet and plasma glucagon levels and also hyperaminoacidemia and α cell hyperplasia, which are consistent responses to absent glucagon signaling; notably, there was only a modest decrease in fasting glucose turnover, suggesting that glucagon action is not needed for normal basal hepatic glucose production. In contrast to the animals with PC1/3 knockdown in which *Pcsk2* expression did not change, eliminating PC2 caused a compensatory increase in *Pcsk1* expression with more than doubling of islet GLP-1 content and an increase in stimulated GLP-1 secretion. Although islet GLP-1 content in *Pcsk2*^αcell−/−^ mice was far lower than the overall content of proglucagon peptides in controls and comparable to the rise in α cells of high-fat diet–fed mice, these animals had a remarkable phenotype. Insulin secretion and glucose tolerance were superior in *Pcsk2*^αcell−/−^ mice relative to controls, indicating that while glucagon may be the physiological mediator of α cell–to–β cell communication, an increase in α cell GLP-1 can enhance the action of this pathway. Previous work has shown that transgenic overexpression of PC1/3 in mouse islets has effects on insulin secretion, similar to what we report here (*50*). Our findings raise the possibility that variation in *Pcsk2* expression, with a reactive change in the activity of *Pcsk1*, adjusts the gain on α cell–to–β cell communication. Understanding the mechanisms whereby α cell PC expression and activity are balanced has important implications for the regulation of insulin secretion.

Deletion of both *Pcsk1* and *Pcsk2* eliminated most of islet glucagon and GLP-1. These animals had glucose intolerance, elimination of amino acid–stimulated insulin secretion, and severe reduction of GSIS. This model mimics our previous description of a mouse line with constitutive deletion of proglucagon and thus adds further support to the importance of α cell–to–β cell communication. The failure of the *Pcsk1:Pcsk2*^αcell−/−^ mice to generate any glucagon or GLP-1 supports earlier studies demonstrating that PC1/3 and PC2 are essential for α cell processing of proglucagon (*51*). The physiological significance can be derived from the impaired glucose tolerance in the *Pcsk1:Pcsk2*^αcell−/−^ mice when challenged with an IPGTT. These knockout mice also displayed elevated sensitivity to GIP, which improved glucose tolerance during an OGTT (an intervention that relies heavily on β cell GIPR signaling). Last, the normal glucose tolerance between *Pcsk1:Pcsk2*^αcell−/−^ and control mice in the setting of an MTT [an intervention that requires both GIPR and GLP-1R signaling (*20*)] indicates that the enhanced GIPR sensitivity is masking the defect in GLP-1R.

In recent years, there has been accumulating evidence to support α cells and glucagon as potent regulators of insulin secretion (*9*, *13*–*16*, *20*). However, the importance of α cell–to–β cell communication has been challenged, both from a conceptual point of view (*52*) and from experiments showing that mice with islets composed only of β cells have intact insulin secretion and glucose tolerance (*53*). The differences in models and approaches in this study (*53*) compared to ours (*9*, *13*, *20*) and those of others (*14*–*16*, *32*) preclude any firm conclusions that would explain the discrepancies in outcomes. Targeted destruction of α cells, using identical mouse models, produced an impaired glucose tolerance phenotype in some studies (*32*) but not others (*53*, *54*). It is possible that deeper phenotyping of these distinct experimental models with the loss of glucagon action would help address these discrepancies.

Last, our observations that GLP-1 levels were substantially higher in human islets raise the possibility that α cell production of GLP-1 may be more important in human islets compared to rodent islets. The variability in GLP-1 content across human islets was broad and not clearly explained by BMI, age, or HbA1c. It is likely that our studies are underpowered to detect potential associations with these parameters and that the impact of these basic subject characteristics is clouded by premorbid pathology. We did find a positive correlation between islet GLP-1 content and insulin secretion. Future work that can extend these correlations toward causation is needed. One possibility is that islet GLP-1 levels increase as an adaptive response to chronic demands. While these were not demonstrated in high-fat diet–fed and STZ-treated mice, this does not rule out adaptive proglucagon processing in humans. Understanding the factors that enable this and whether this can support enhanced insulin secretion to overcome the metabolic stress are important questions to address the role of α cells in metabolism.

In summary, we present data that definitively show both rodent and human islets process proglucagon with PC1/3 to generate bioactive GLP-1 (summarized in fig. S7). In mice, the GLP-1 made by α cells is dispensable, as α cell–to–β cell communication is mediated by the relatively higher levels of glucagon. However, deletion of glucagon production shows the importance of α cell–derived GLP-1, reinforcing that GLP-1 can support α cell–to–β cell communication. In human islets, the GLP-1:glucagon ratio is much higher than that in mouse islets, with the average GLP-1 content being ~50% that of glucagon and several sets of islets having more GLP-1 than glucagon. Understanding the implications of this observation and the factors that drive higher GLP-1 production in human islets is a requisite next step in this journey. Together with our mouse data, this emphasizes the importance of α cell–to–β cell communication for insulin secretion and glucose tolerance.

## MATERIALS AND METHODS

### Reagents

STZ (Sigma-Aldrich) was prepared in 0.1 M citrate buffer (pH 4.5). Stable isotope–labeled (heavy, 3305.67 monoisotopic molecular weight) and unlabeled (light) GLP-1(7–36)NH_2_ were custom synthesized by Thermo Fisher Scientific as AQUA Ultimate-grade peptides (>97% purity, >99% isotope enrichment). The dipeptidyl peptidase 4 (DPP4) inhibitor (diprotin A) was purchased from Bachem. 6,6-*D*_2_-Glucose was from Cambridge Isotope Laboratories. The GLP-1R antagonist Ex9 was purchased from GenScript. Mouse [D-Ala2] GIP (Novo Nordisk) was reconstituted in phosphate-buffered saline (PBS). The glucagon receptor antibody was previously described (*46*) and provided by Eli Lilly and Company for use in these studies.

### Human islets

Human islets were purchased from the Alberta Diabetes Institute IsletCore at the University of Alberta. The sample preparation of the donors obtained approval from the Human Research Ethics Board of the University of Alberta. All donors’ families gave informed consent for the use of pancreatic tissue in research. Islet insulin secretion experiments were performed by Alberta Diabetes Institute IsletCore (humanislets.com).

### Animals

All animals were maintained and used in accordance with protocols approved by the Duke University Institutional Animal Care and Use Committee (protocol no. A202-23-10). Mice were housed under a 12-hour light/dark cycle and provided free access to food and water. C57BL6/J background mice with LoxP sites in the *Pcsk1* or *Pcsk2* allele (*55*, *56*) were crossed with *Gcg-CreERT2* mice on the same background (*57*) to generate α cell–specific mouse lines*.* Mice with floxed alleles served as controls. Tamoxifen (50 mg/ml; Sigma-Aldrich) was administered in 8-week-old mice by oral gavage for four consecutive days to all mice*. Gcg^−/−^* mice have been previously described (*6*). For high-fat diet feeding experiments, mice were fed a high-fat diet containing 60 kcal % of calories as fat (Research Diets). For STZ treatment, mice received a daily injection of STZ (50 mg/kg, intraperitoneally) for 5 days. B6.Cg-Lepob/J (*ob/ob*) mice were purchased from the Jackson Laboratory (strain no. 000632). Only male mice were used for in vivo studies, while both sexes were used for ex vivo analyses.

### Islet isolation

Primary islets were isolated from mice according to previously published methods (*9*). The pancreas was inflated by injecting collagenase V (0.7 mg/ml; Sigma-Aldrich) in Hanks’ balanced salt solution. Islets were recovered overnight in RPMI (Gibco) with 10% fetal bovine serum (Gibco) before all experiments.

### GLP-1 measurement

Standard curves were generated in acid ethanol (70% ethanol, 1.5% HCl, and DPP4 inhibitor) extracts from 200 *Gcg^−/−^* mice (*6*) to which 3000 fmol of heavy GLP-1 and varying concentrations of authentic (“light”) GLP-1(7–36)NH_2_ were added. Islet unknowns used 200 mouse or human islets extracted in acid ethanol with 3000 fmol of heavy GLP-1. Standards and unknowns were subjected to solid-phase extraction using Sep-Pak tC18 cartridges (Waters), and the eluate was dried in a speed vacuum. Samples were resuspended in 500 μl of 0.1% trifluoroacetic acid for the measurement of total peptide quantity using the Pierce colorimetric peptide assay (Thermo Fisher Scientific). Samples were then redried in a speed vacuum, and each sample was resuspended in 2% acetonitrile/0.1% formic acid (FA) at 0.5 μg/μl. Samples were analyzed by nanoflow liquid chromatography (nLC) on an EASY-nLC 1200 UHPLC, followed by electrospray ionization with an EASY-Spray source and tandem MS (MS/MS) with a Q Exactive Plus Hybrid Quadrupole-Orbitrap (Thermo Fisher Scientific). Each sample injection of 1 μl (0.5 μg) was first trapped on an Acclaim PepMap 100 C18 trapping column (3-μg particle size, 75 μm by 20 mm) with solvent A (0.1% FA) at a variable flow rate (maximum pressure of 500 bars), followed by analytical separation over a 25-min gradient (flow rate of 400 nl/min) of 5 to 35% solvent B (90% acetonitrile and 0.1% FA) using an Acclaim Pep-Map RSLC C18 analytical column (2-μg particle size, 50-μm by 150-mm column) with a column temperature of 55°C. MS2 spectra (product ions) were collected by parallel reaction monitoring using an inclusion list for the following precursors: HAEGTFTSDVSSYLEGQAAKEFIAWLVKGR-amide [light GLP-1; 824.9229 mass/charge ratio (*m*/*z*); charge state, 4], HAEGTFTSDVSSYLEGQAAKEFIAWLVKGR-amide (heavy GLP-1; 827.4297 *m*/*z*; charge state, 4), and HSQGTFTSDYSKYLDSRRAQDFVQWLM[+15.995]NT (light glucagon; 875.1599 *m*/*z*; charge state, 4). Targeted MS2 scans were collected using 140,000 resolution (r), automatic gain control (AGC) target of 2 × 10^5^ ions, 500-ms maximum injection time (max IT), loop count of 3, 1.6 *m*/*z* isolation window, and normalized collision energy (NCE) of 29. For every five parallel reaction monitoring scans of each precursor (15 total MS2 scans), a full-scan MS1 spectrum was collected using 140,000 r, AGC target of 1 × 10^6^ ions, and max IT = 500 ms. Raw files were analyzed using Skyline-daily (*58*), as described previously (*59*). Extracted ion chromatograms were generated from a minimum of four MS2 fragment (product) ions matching within 0.05 *m*/*z* to the targeted peptide, filtering for the most intense ions from the library spectrum. For all standard and experimental samples, the area under the curve (AUC) for light GLP-1 was normalized to the heavy GLP-1 internal standard. Standard curves were generated with triplicate analysis for each amount of added “light” GLP-1, with linear regression using 1/×2 weighting. The calculated absolute abundance of endogenous GLP-1 in each sample, along with the light/heavy ratios and peak area dot products (dotp), was exported in Excel. As a qualitative assessment of endogenous light glucagon, AUC was normalized to the total ion current from MS1 scans.

The coefficient of variation for all standard curve points measured in triplicate was below 20% at concentrations above 50 fmol/μg. For standard curves where 50 fmol/μg was the lowest concentration of light GLP-1, the blank sample (0 fmol/μg) from *Gcg^−^/^−^* islets produced a signal equivalent to ~10 fmol/μg on the basis of the standard curve fit (which was not constrained to pass through zero), suggesting low-level background interference. The ratio of dot products (rdotp), comparing the relative abundances of fragment ions in the experimental data to those in a reference library spectrum, was used as a metric of specificity. Confident identifications of light and heavy peptide pairs required rdotp values >0.9 for both, along with manual verification of coelution. For plasma samples and islet extracts, active and total GLP-1 was measured by the ELISA kit (Meso Scale), as previously described (*60*).

### Flow cytometry

A total of 150 to 180 islets was handpicked into 1.5-ml tubes and rinsed once in PBS before incubation with Accutase (Sigma-Aldrich) for 12 min at 37°C. Dispersed islet cells were acquired in a fluorescence-activated cell sorting cytometer (Beckman-Coulter MoFlo Astrios). Forward scatter and side scatter were used to separate single cells from debris and doublets, and cells were separated by autofluorescence and side scatter into enriched populations of α, β, and δ cells, as previously described (*61*).

### mRNA analysis

Whole islets and enriched populations of sorted cells were collected into TRIzol (Sigma-Aldrich) for RNA extraction. cDNA was synthesized from 100 ng of RNA with the reverse transcription kit (Thermo Fisher Scientific). For liver, gut, and hindbrain tissues, 20 to 25 mg of tissues was collected into TRIzol and extracted by a tissue lyser (Qiagen). cDNA was synthesized from 1000 ng of RNA. Quantitative polymerase chain reaction was run using TaqMan reagents on an Applied Biosystems QuantStudio Dx Real-Time thermal cycler (Thermo Fisher Scientific). The primer sequences are listed in table S1.

### In vivo physiological tolerance tests

Mice were acclimated to housing, handling, and injections for 1 to 2 weeks and fasted for 5 hours before in vivo testing. Following baseline blood glucose sampling, glucose was administered at a dose of 1.5 g/kg in PBS intraperitoneally or orally, Ensure was administered orally at 10 ml/kg, and blood was sampled from the tail at standard intervals over 2 hours. For the measurement of hormones, blood was collected from the tail vein in EDTA-coated capillary tubes, and plasma was separated by centrifugation at 12,000*g* for 15 min. Insulin was measured by ELISA (Mercodia). For insulin tolerance tests, basal blood was taken, an insulin analog (Humalog) was injected intraperitoneally at a dose of 1.5 U/kg, and blood was sampled from the tail at standard intervals over 90 min. Alanine/glycerol tests were conducted in mice after an overnight fast and intraperitoneal injection of alanine (0.75 g/kg) or glycerol (0.83 g/kg). Epinephrine (200 μg/kg; Sigma-Aldrich) challenges were performed in mice after a 5-hour fast by intraperitoneal injection. For all in vivo testing, blood glucose was measured using a glucometer (Contour Next EZ).

### Islet perifusion

Islet perifusion was performed as previously described (*9*). Briefly, equal numbers of islets (70 islets) were handpicked and placed into chambers containing 2.7 mM glucose KRPH buffer (140 mM NaCl, 4.7 mM KCl, 1.5 mM CaCl_2_, 1 mM NaH_2_PO_4_, 1 mM MgSO_4_, 2 mM NaHCO_3_, 5 mM Hepes, and 0.1% FA-free bovine serum albumin; pH = 7.4) with 100 μl of Bio-Gel P-4 Media (Bio-Rad). Islets were perifused at a constant rate of 200 μl/min in 2.7 mM glucose KRPH buffer for 48 min and then perifused on the basis of the experimental conditions. All treatments were prepared in KRPH buffer. Insulin was assayed with a Lumit Insulin kit (Promega) using an EnVision plate reader (PerkinElmer).

### Amino acid measurements

Plasma samples were collected from mice following a 5-hour fast and analyzed by liquid chromatography–MS/MS (LC-MS/MS) using a Quattro Micro instrument (Waters Corporation), as previously described (*42*).

### Glucagon measurement

Islet glucagon was measured by LC-MS/MS in ~100 mouse islets extracted in acid ethanol (70% ethanol, 1.5% HCl, and DPP4 inhibitors). Plasma was collected into EDTA-coated capillary tubes containing DPP4 inhibitor/aprotinin (Sigma-Aldrich). LC-MS/MS used monoclonal antibodies to enrich glucagon, as previously described (*62*). Plasma glucagon was measured by ELISA (Mercodia).

### Immunohistochemistry

Pancreas samples from 17- to 24-week-old mice were weighed, laid flat in cassettes, and fixed in 10% formalin solution before being embedded in paraffin. Pancreatic sections were cut at a 5-μm thickness and costained with insulin (CST), proglucagon (CST), or glucagon (Sigma-Aldrich) antibodies. The images were scanned by Aperio Image Scope software at 20× view. Digital images were analyzed with ImageJ software (version 1.46R, National Institutes of Health) for α cell mass.

### Glucose turnover

Carotid and jugular cannulation surgery was performed 1 week before the experiment. Briefly, mice were anesthetized with isoflurane, the catheters were placed in the left carotid artery, and the right external jugular vein and catheters were connected to a vascular access port. Patency and body weight were monitored daily for the first 7 days postsurgery. Mice were fasted for 3 hours in infusion cages to allow for acclimation. Twenty minutes before tracer infusion, animals were attached to a sterile saline-primed loop-wire tether and swivel system (Instech Laboratories Inc., PA) and allowed 20 min to acclimate. 6,6-*D*_2_-Glucose in a sterile 0.9% sodium chloride solution was loaded onto double-barrel Harvard Apparatus pumps (Instech Laboratories Inc., PA) and connected to the swivel. At the baseline, glycemia was measured, and whole-blood samples were collected. Continuous infusions started at 9.84 mg kg^−1^ min^−1^ for 5 min to prime the endogenous glucose pool. After 5 min, the infusion rate was adjusted to 0.492 mg kg^−1^ min^−1^ for 115 min to reach the desired tracer-to-tracee ratio enrichment of 3%. Whole blood for tracer enrichment and glycemia was collected at 120, 130, 140, and 150 min into tracer infusion. After 150 min, epinephrine was continuously infused at a rate of 5 μg kg^−1^ min^−1^ to elevate blood glucose levels for a total of 60 min. Whole blood for tracer enrichment and glycemia was collected at 40, 50, and 60 min after the start of the intervention. Plasma enrichment of 6,6-*D*_2_-glucose was measured by LC-MS, as previously reported (*63*). Analysis and calculations were performed as previously described (*35*).

### Statistical tests

Data are presented as the means ± SEM with values for individual animals or assays shown in figures. Data were analyzed using *t* tests and analysis of variance (ANOVA), as indicated in figure legends. A *P* value of less than 0.05 was considered statistically significant.
