# Supplementary material for: α cells use both PC1/3 and PC2 to process proglucagon peptides and control insulin secretion
Source: Sci Adv. 2025 Sep 19;11(38):eady8048. doi: 10.1126/sciadv.ady8048 (PMC12448133; doi:10.1126/sciadv.ady8048)
Supplement: Supplementary file 1 — Figs. S1 to S7 Tables S1 and S2 [file sciadv.ady8048_sm.pdf]

Science Advances
AAAS

Supplementary Materials for

α cells use both PC1/3 and PC2 to process proglucagon peptides and control insulin secretion

Canqi Cui *et al.*

Corresponding author: Jonathan E. Campbell, jonathan.campbell@duke.edu

Sci. Adv. **11**, eady8048 (2025)
DOI: 10.1126/sciadv.ady8048

This PDF file includes:

Figs. S1 to S7
Tables S1 and S2

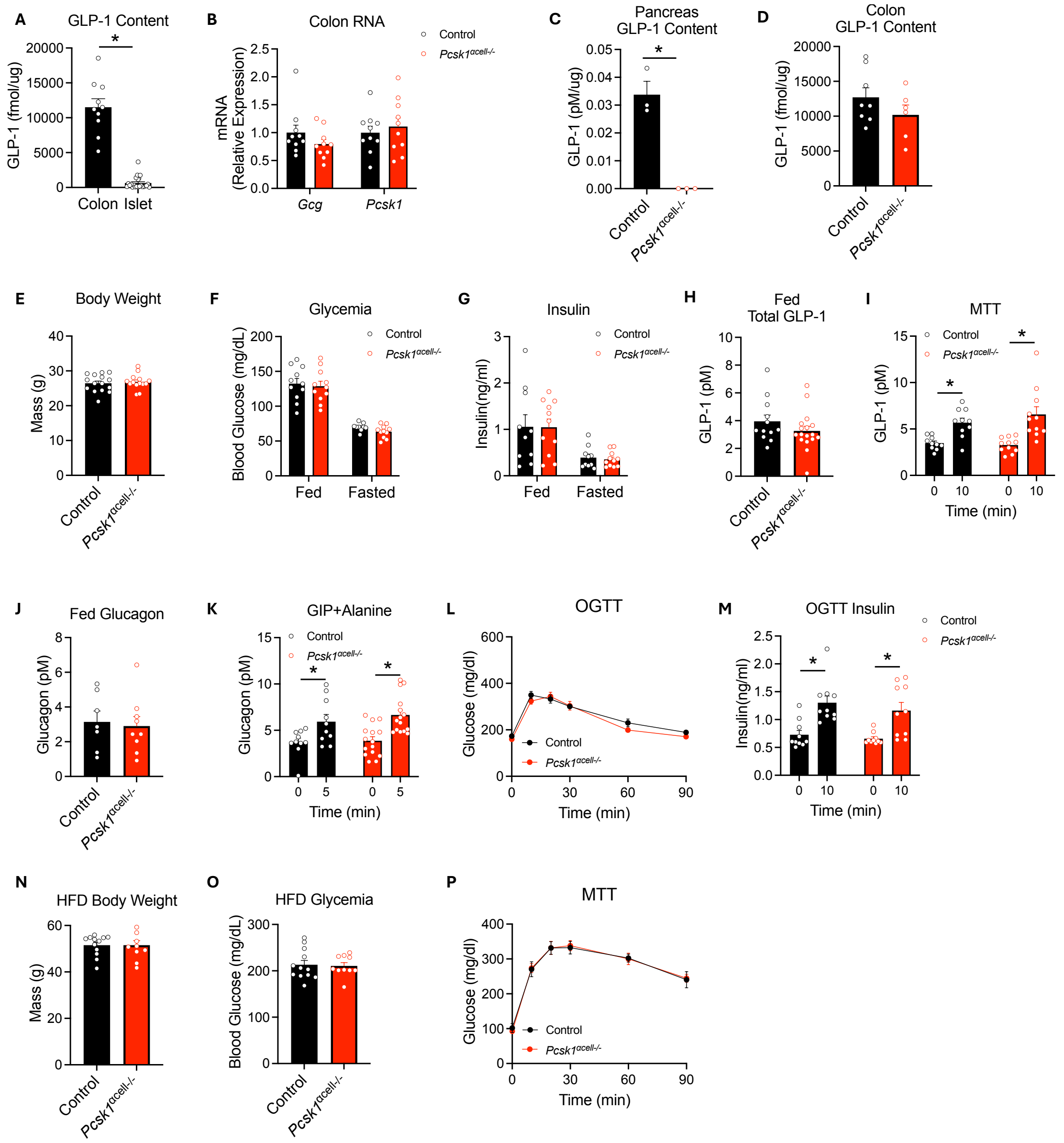

Supplemental Figure 1: (A) Mouse tissue GLP-1(7-36)NH2 content measured by mass spectrometry (n= 14, 29). (B) Relative mRNA expression in colon (n=10). (C) Tissue active GLP-1(7-36)NH2 content in colon measured by mass spectrometry (n=8,6). (D) Tissue active GLP-1 content in pancreas tissue measured by ELISA (n=3). (E) Body weight of 12-week-old mice on chow diet (n=15, 14). (F-G) Ab libitum and overnight fasted (F) glycemia and (G) plasma insulin concentrations (n=10, 11). (H-I) Plasma total GLP-1 levels measured ab libitum (n=12, 17) and during a mixed nutrient tolerance test (MTT, ensure) in high-fat fed mice (n=10). (J-K) Plasma glucagon levels in lean mice measured (J) ab libitum (n=7, 9) or in response to GIP (4 nmol/kg) and alanine (0.325 g/kg) (n= 9, 15). (L-M) (L) Glycemia and (M) insulin concentrations during an oral glucose tolerance test (OGTT, 1.5 g/kg) in lean mice (n = 12, 12). (N-P) (N) Body weight, (O) ad libitum glycemia, and (P) glycemia during a MTT in mice on high-fat diet for 14-weeks (n=12,10). *-P<0.05, as indicated. Data are shown as mean±SEM. Statistical analysis were done with a student's unpaired t-test (A-H, J, N, and O) or ANOVA with post-hoc analysis (I, K, L, M, and P)

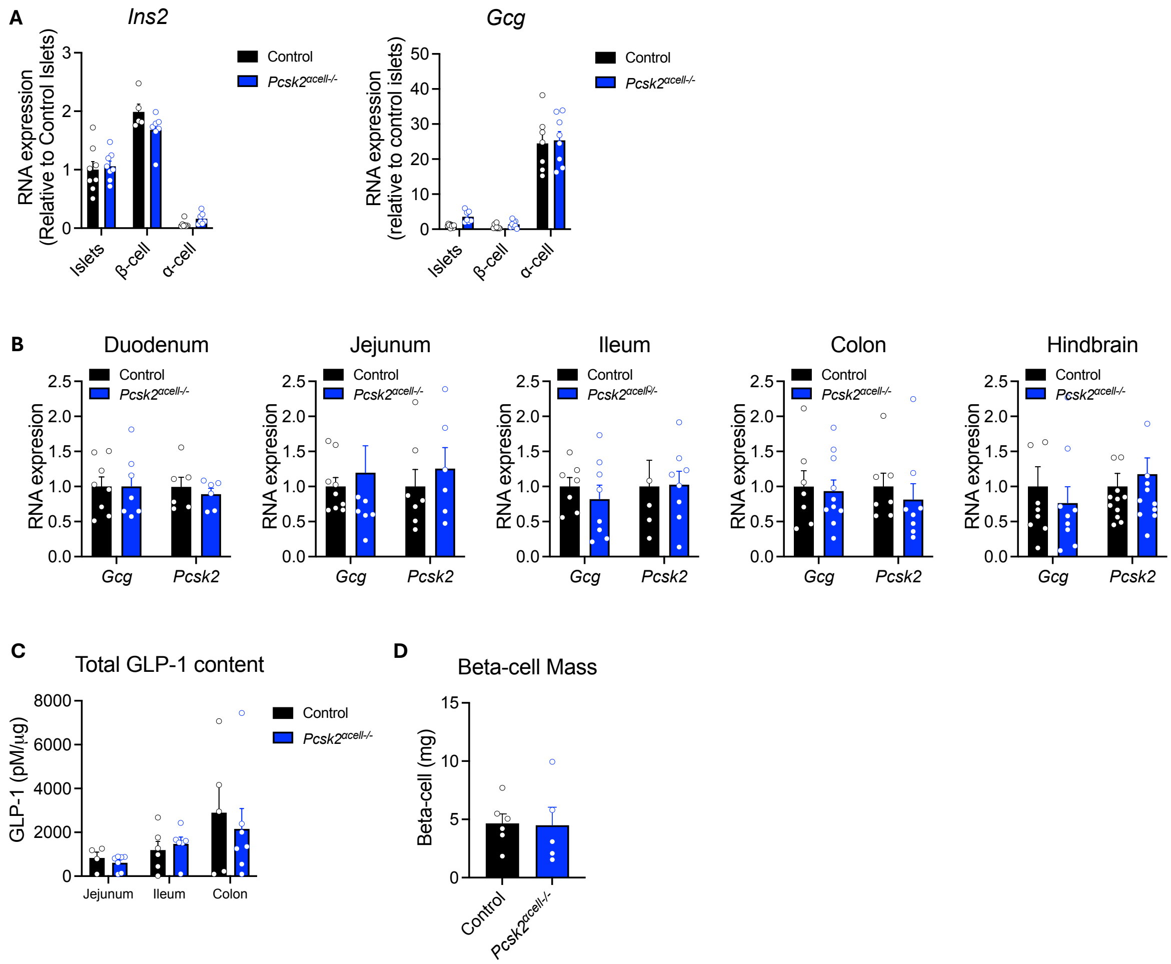

Supplemental Figure 2: (A) RNA expression in islets and enriched populations of sort alpha- and beta-cells (n=10, 8). Data are normalized to gene expression of control values measured in whole islets. (B) RNA expression in intestinal tissues (n=8-9) and hindbrain (n=10-12). (C) Total GLP-1 levels in intestinal tissues measured by ELISA (n=4-7). (D) β-cell mass in in 20-week-old mice (n=5). Data are shown as means ± SEM. Statistical analysis were done with a student's unpaired t-test.

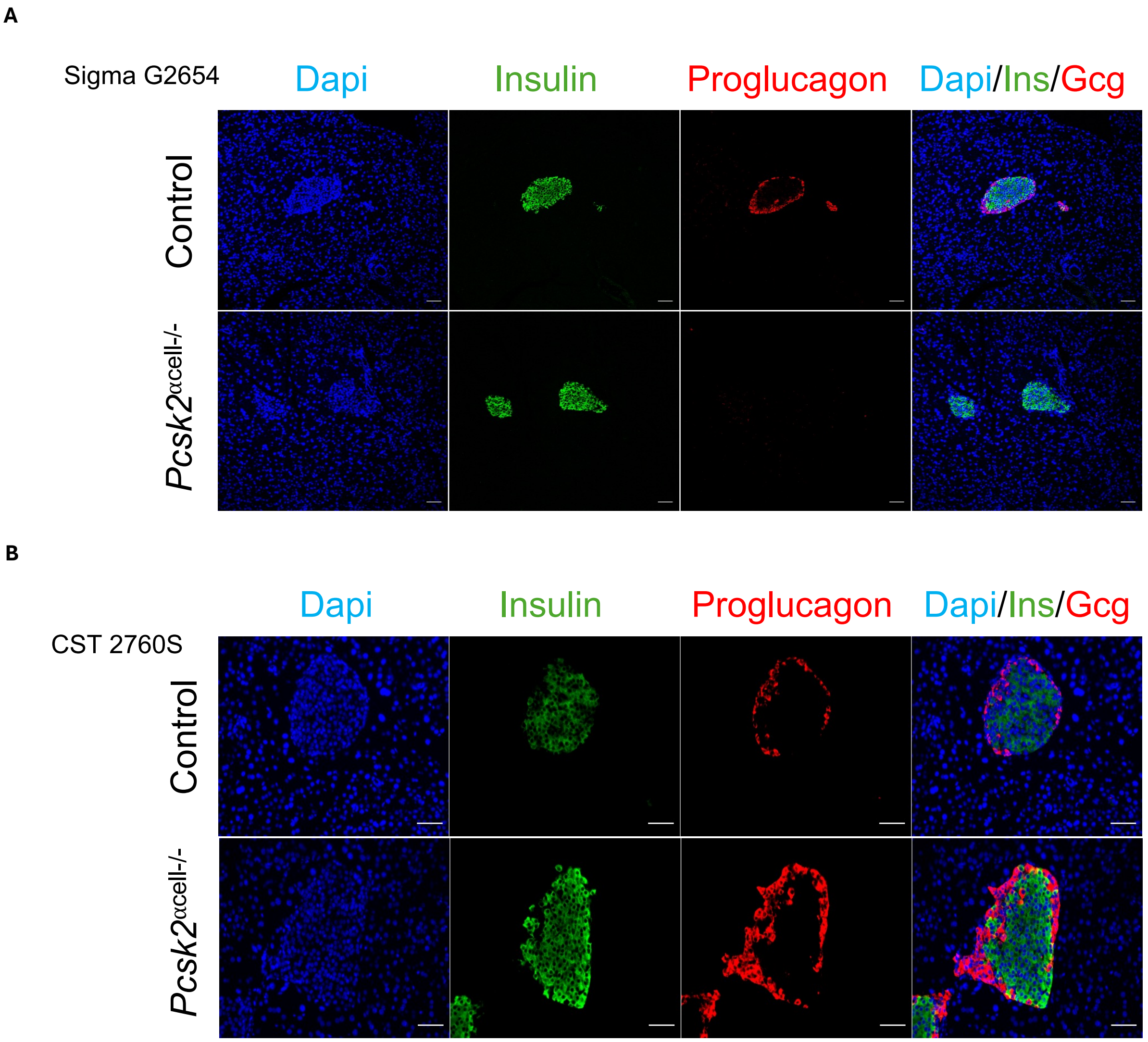

Supplemental Figure 3: Representative images for immunostaining in pancreas samples. (A) Samples are stained for DAPI, insulin, and an antibody that detects mature, processed glucagon. (B) Samples are stained for DAPI, insulin and an antibody that detects proglucagon. Scale bar, 75μm.

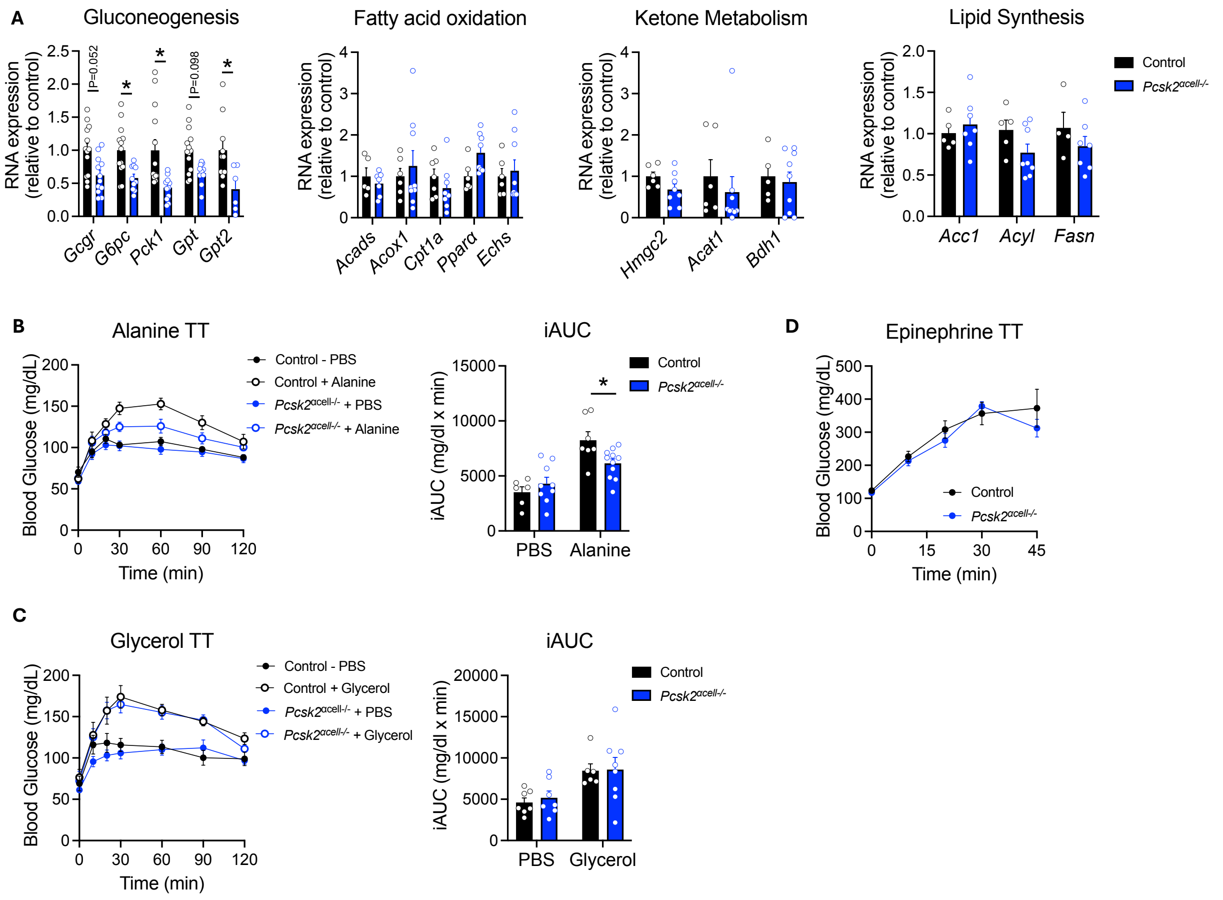

Supplemental Figure 4: (A) RNA expression in liver tissue collected from fasted, lean mice (n=14). (B) Alanine tolerance test in 16 hour fasted mice (n=9, 12). (C) Glycerol tolerance test in 16 hour fasted mice (n=10). D) Epinephrine tolerance test in 5-hour fasted mice. (n=11). *-P<0.05, as indicated. Data are shown as mean±SEM. Statistical analysis were done with a student's unpaired t-test (A) or ANOVA with post-hoc analysis (B-D).

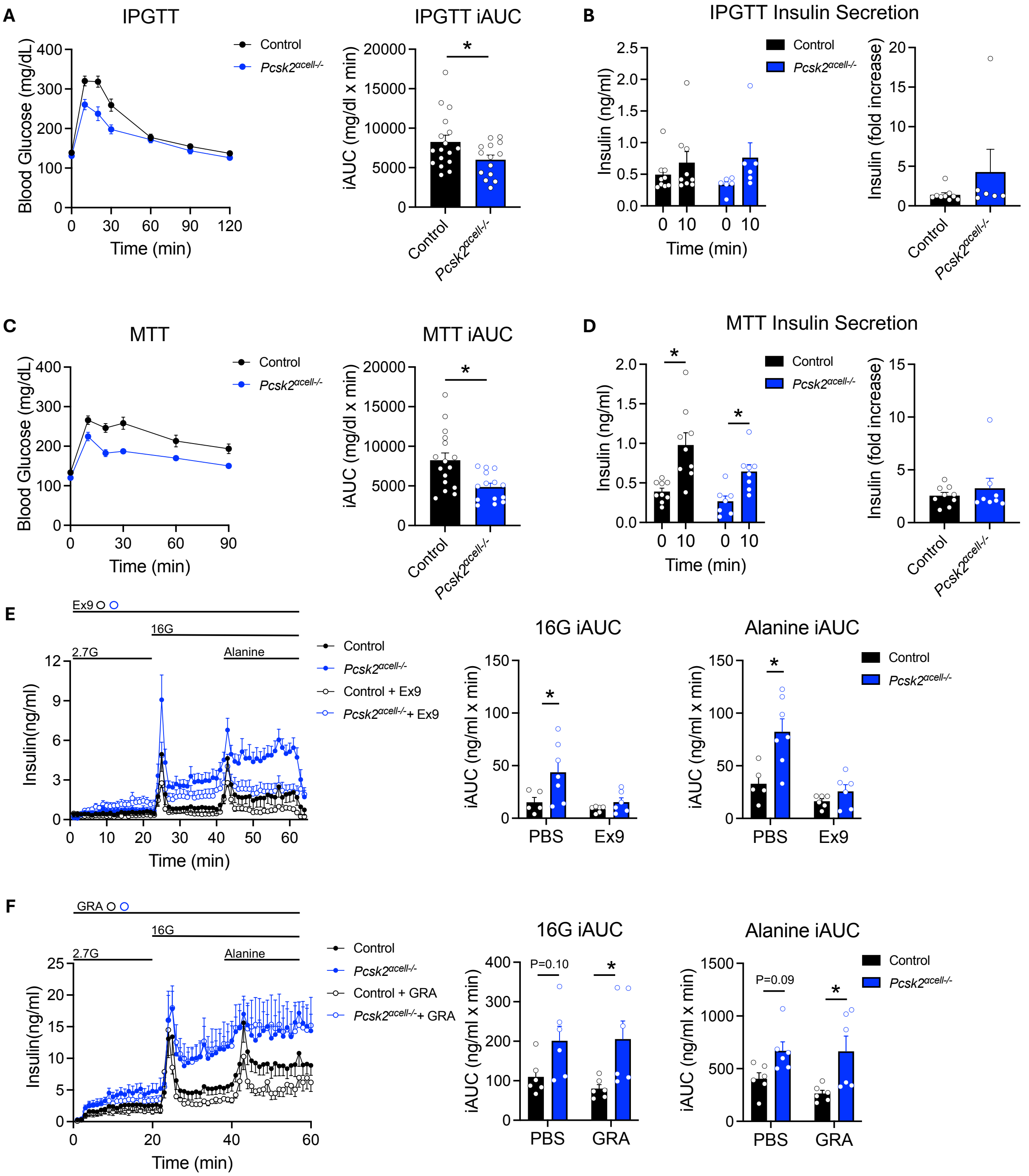

Supplemental Figure 5: (A, B) (A) Glycemia (n=15, 14) and (B) plasma insulin (n=14, 10) concentrations during an intraperitoneal glucose tolerance test (IPGTT, 1.5 g/kg) in 5 hour fasted mice. (C-D) Glycemia (n=15, 16) and (B) plasma insulin concentrations during an mixed nutrient tolerance test (ensure) in 5 hour fasted mice (n=9, 8). (E-F) Insulin secretion in perifused islets stimulated in the presence of (E) exendin(9-39) (Ex9) or (F) a glucagon receptor antagonist (GRA). iAUCs are calculated for 16 mM glucose alone (min 16-32) and 16 mM glucose with 3 mM alanine (min 40-60). *-P<0.05, as indicated. Data are shown as mean±SEM. Statistical analysis were done with a student's unpaired t-test (A, B, C) or ANOVA with post-hoc analysis (A-F).

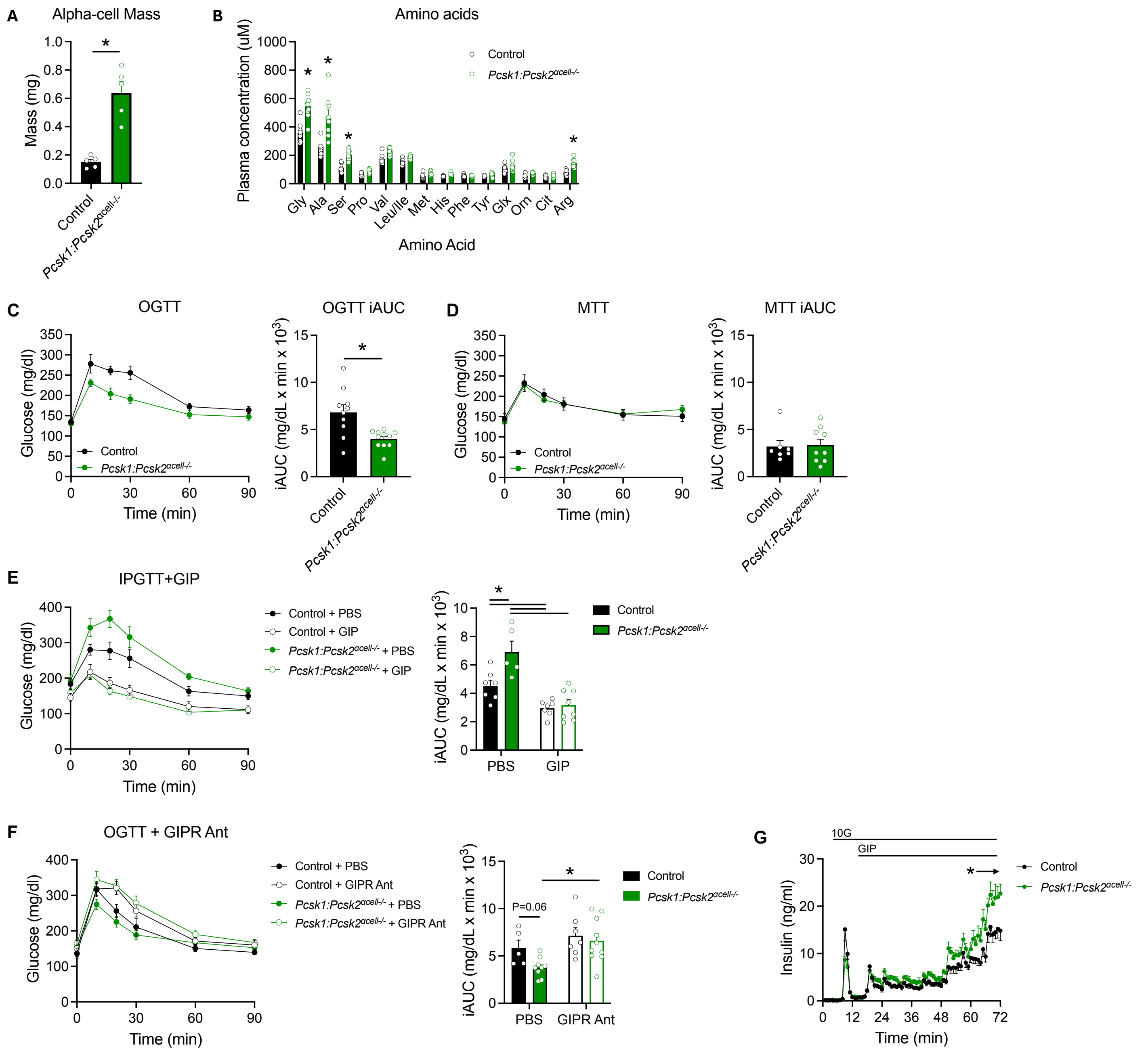

Supplemental Figure 6: (A) Alpha-cell mass in 18-20 week old mice (n=5). (B) Fasted plasma amino acid levels in 18-20 week old mice (n=8,9). (C) Oral glucose tolerance test (OGTT) in 12-14 week old mice. (D) Mixed meal tolerance test (MTT) in 12-14 week old mice. (E) Intraperitonel glucose tolerance test (IPGTT) where PBS or GIP was given 10 minutes prior to glucose. (F) OGTT where a GIPR antagonist (*67*) was given 1 hour prior to glucose. (G) Perifused islets at 10 mM glucose and with a GIP ramp (0-10 nM). *-P<0.05, as indicated. Data are shown as mean±SEM. Statistical analysis were done with a student's unpaired t-test (A-D) or a two-way ANOVA (E-G).

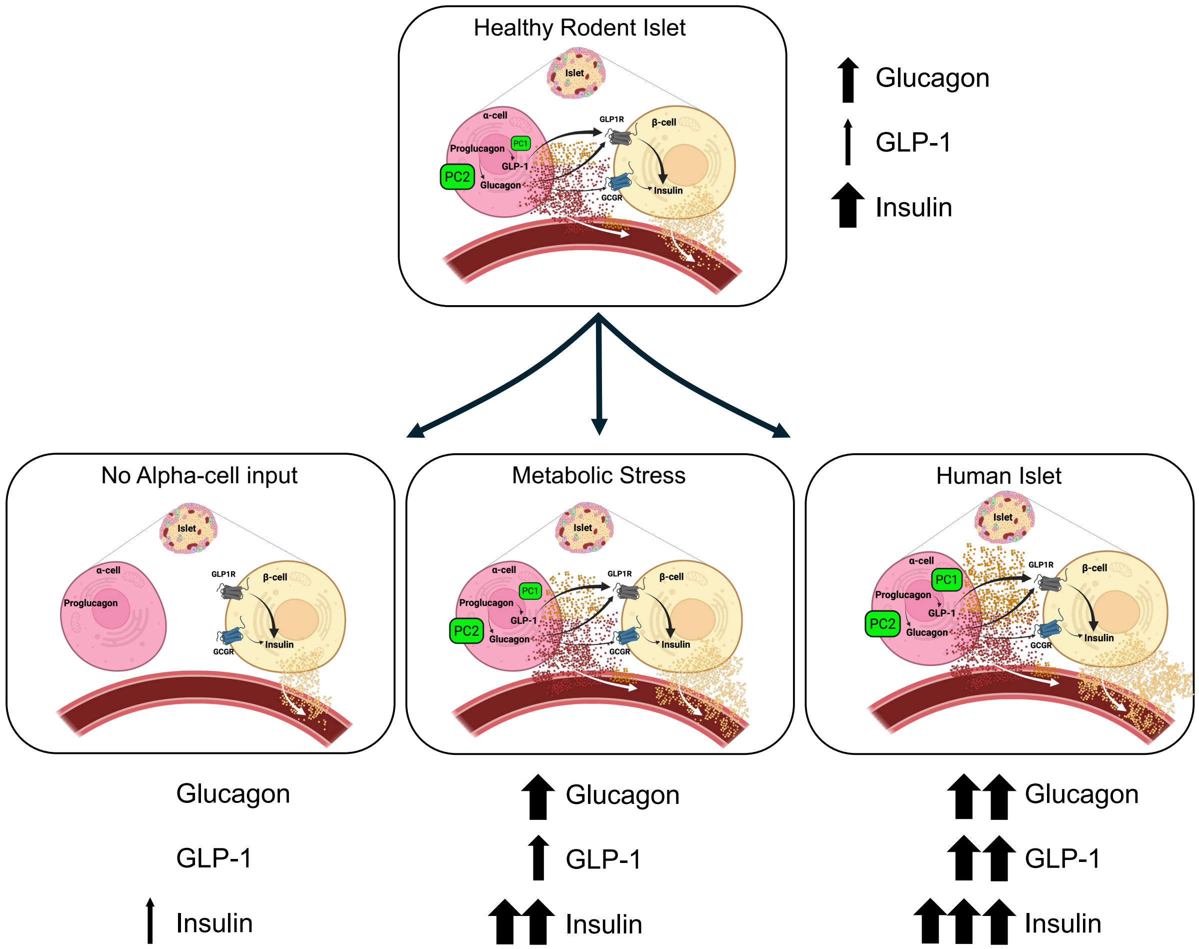

Supplemental Figure 7: A schematic illustrating alpha- to beta-cell communication in various islet states. In healthy rodent islets, glucagon is the predominant proglucagon peptide that facilitates beta-cell function. Without alpha- to beta-cell communication, the rate of insulin secretion is decreased dramatically, leading to glucose intolerance. Various metabolic stressors increase PC1/3 expression and the production of GLP-1 from alpha-cells. However, glucagon continues to be the dominant proglucagon peptide that facilitates alpha-to beta-cell communication. Human islets have variable levels of GLP-1 production, with some being equivalent to rodent islets and some being substantially higher. It is unclear which factors are responsible for the marked heterogeneity in human islets, and whether this shifts the reliance on GLP-1 for alpha- to beta-cell communication. There is a positive correlation between GLP-1 content and the rates of insulin secretion in human islets.

Gene	Name	Taqman Assay ID
Acads	Acyl-Coenzyme A dehydrogenase, short chain	Mm00431617_m1
Acat1	Acetyl-Coenzyme A acetyltransferase 1	Mm00507463_m1
Acox1	Acyl-Coenzyme A oxidase 1, palmitoyl	Mm01246834_m1
Bdh1	3-hydroxybutyrate dehydrogenase, type 1	Mm00558330_m1
Cpt1a	Carnitine palmitoyltransferase 1a, liver	Mm01231183_m1
Echs	Enoyl Coenzyme A hydratase, short chain, 1, mitochondrial	Mm01276347_m1
G6pc	Glucose-6-phosphatase catalytic subunit 1	Mm00839363_m1
Gcg	Glucagon	Mm01269055_m1
Gcgr	Glucagon receptor	Mm00433546_m1
Gpt	Glutamic pyruvic transaminase	Mm00805379_g1
Gpt2	Glutamic pyruvic transaminase2	Mm00558028_m1
Hmgcs2	3-hydroxy-3-methylglutaryl-Coenzyme A synthase 2	Mm00550050_m1
Pck1	Phosphoenolpyruvate carboxykinase 1	Mm01345254_m1
Pcsk1	Proprotein convertase subtilisin/kexin type 1	Mm00500976_m1
Pcsk2	Proprotein convertase subtilisin/kexin type 2	Mm01247058_m1
Pparα	Peroxisome proliferator activated receptor alpha	Mm00440939_m1
Ppia	Peptidylprolyl isomerase A	Mm02342430_g1

Supplemental Table 1. List of Taqman assay primers

	ID	Age	Sex	BMI	A1c(%)	Diabetes	GLP-1 (fmol/ug)
1	R088	20	Male	40.9	5.8	non-T2D	2836.9
2	R203	50	Male	44.37	5.7	non-T2D	8295.3
3	R399	47	Male	40.3	5.5	non-T2D	10152.9
4	R446	64	Male	40.7	6.7	non-T2D	10362.6
5	R193	69	Male	39.48	6.2	non-T2D	3436.6
6	R217	71	Female	35.5	6.3	non-T2D	2294.4
7	R290	74	Female	35.8	5.9	non-T2D	4376.9
8	R436	57	Female	39.7	3.7	non-T2D	18564.1
9	R448	61	Female	36.1	5.8	non-T2D	6748.6
10	R426	33	Female	31.9	5.4	non-T2D	49592.7
11	R376	20	Male	26.2	11.7	T1D	4241.8
12	R410	59	Female	26.4	4.8	non-T2D	26017.3
13	R423	28	Male	25.7	5.3	non-T2D	7363.7
14	R424	27	Male	27.5	5.7	non-T2D	25727.5
15	R296	50	Female	23.9	6.3	T1D	22237.1
16	R380	29	Male	19	5.1	T1D	7615.9
17	R475	51	Female	31.6	4.6	non-T2D	3888.8288
18	R415	32	male	26.6	5.9	non-T2D	4162.00
19	R418	44	Male	34.9	5.4	non-T2D	10465.00
20	SAMN27361472	34	Male	31.8	/	non-T2D	7200.30
21	R440	35	Female	26.7	3.8	non-T2D	14274.44
22	R430	49	Male	24.4	4.8	non-T2D	20661.99
23	SAMN26527277	61	Male	22.6	/	non-T2D	23176.83
24	R445	33	Male	24.2	5.6	non-T2D	1229.99
25	R442	53	Female	12.8	5.7	non-T2D	12317.75
26	SAMN28867622	36	Male	29.6	/	non-T2D	8142.64
27	SAMN30686018	39	Male	32.4	/	non-T2D	2080.01
28	R170	49	Male	38.6	6.5	T2D	1750.2
29	R213	57	Male	23.14	6.7	T2D	2464.7
30	R222	62	Male	25.95	10	T2D	10912.1
31	R236	51	Male	35.3	8.6	T2D	108.3
32	R240	55	Female	30.91	6.9	T2D	49375.8
33	R241	65	Male	21.8	9.9	T2D	2016.6
34	R259	45	Female	33.1	6.4	T2D	18017.5
35	R276	54	Female	24.4	7.2	T2D	51893.4
36	R402	57	Male	26.7	6	T2D	2714.5
37	R452	46	Male	34.9	6.1	T2D	13328.2
38	R432	66	Female	36.5	8.6	T2D	18549.3521

Supplemental Table 2. Islet donor characteristics
